# Particulate matter in the cultivation area may contaminate leafy vegetables with heavy metals above safe levels in Korea

**DOI:** 10.1007/s11356-019-05825-4

**Published:** 2019-07-02

**Authors:** Kyungdeok Noh, Luc The Thi, Byoung Ryong Jeong

**Affiliations:** 1 Department of Horticulture, Division of Applied Life Science (BK21 Plus Program), Graduate School, Gyeongsang National University, Jinju, 52828 Republic of Korea; 2 Civilian Headquarter for Solution of Particulate Matter Pollution, Seoul, 06764 Republic of Korea; 3 Institute of Agriculture and Life Science, Gyeongsang National University, Jinju, 52828 Republic of Korea; 4 Research Institute of Life Science, Gyeongsang National University, Jinju, 52828 Republic of Korea

**Keywords:** Air pollutant, PM2.5, Plant, Food safety, Particulate matter pollution

## Abstract

Among air pollutants, particulate matter (PM) has been identified as a major cause of environmental pollutants due to the advancement of industrial development and the generation of smaller particles. Particulate matter, in particular, is defined only by the size of particles and thus is not enough to study its composition yet. However, edible crops grown in contaminated atmospheres can be contaminated with heavy metals contained in particulate matter in the atmosphere, which can seriously damage food safety. In this study, we investigated the influence of the accumulation of particulate matter on leafy vegetables cultivated at areas with different levels of PM in atmosphere. Four districts of Gyeongsangnam-do were chosen to conduct this experiment: outdoor spaces of three respectively located in industrial, near-highway, and rural areas were considered, and research plant growth chambers at Gyeongsang National University were used as the control. After 3 weeks of cultivation in those conditions, the results showed that Pb in milligrams per kilogram of fresh weight (FW) was 0.383 in *Chrysanthemum coronarium* and 0.427 in *Spinacia oleracea* that were grown near the highway, which exceeded the 0.3 mg kg^−1^ FW standard set by the Republic of Korea, EU, and CODEX. However, when those vegetables were sufficiently washed with tap water, it was confirmed that the heavy metal content fell into the safety standard range.

## Introduction

Air pollution is one of the greatest risk factors of our times (Kim et al. 2015b; Li et al. 2016). One of the key indicators of the “better life index” in OECD countries is the concentration of particulate matter (PM10 and PM2.5) (Mizobuchi 2014). The concentration of PM2.5, designated by the World Health Organization as group 1 carcinogen, varies depending on the country and region (Han et al. 2016; Jeevanandam et al. 2018; Kim 2018; Jankowski et al. 2019).

The PM is a widespread air pollutant, consisting of a mixture of solid and liquid particles suspended in air and groundwater (Mohankumar et al. 2016; Park et al. 2018). Even with the same mass concentration depending on the composition of the PM (Lanzerstorfer 2017), the effect on the human body varies considerably (Mukherjee and Agrawal 2017). The physical and chemical characteristics of PM vary with the location (Tong et al. 2016; Bi et al. 2018; Choi et al. 2018; Turkyilmaz et al. 2018). Common chemical constituents of PM include sulfates (Hotze et al. 2010); nitrates; ammonium; other inorganic ions such as ions of Ca, Cl, K, Mg, and Na; organic and elemental carbon; crustal material; particle-bound water; metals (including Cd, Cu, Ni, V, and Zn); and polycyclic aromatic hydrocarbons (PAH) (Nayak 2002; Shrivastava et al. 2018; Weerakkody et al. 2017, 2018a). In addition, biological components such as allergens and microbial compounds are found in PM (He et al. 2016; Schweitzer et al. 2018).

Related studies with plants and particulate matter can be divided into three main categories. The first is how plants reduce particulate matter (Leonard et al. 2016). The PM can either be directly emitted into the air (primary PM) or be formed in the atmosphere from gaseous precursors (Crouse et al. 2016) such as sulfur dioxide, oxides of nitrogen, ammonia, and non-methane volatile organic compounds (secondary PM) (Fu et al. 2016; Guo et al. 2017; Seo et al. 2017). Plants can reduce the PM concentration (Gajbhiye et al. 2016) by directly reducing the primary PM (Chen et al. 2016) by attaching it to their wax layers (Ugolini et al. 2013; Weerakkody et al. 2018b; Liu et al. 2019; Shao et al. 2019) or by reducing the precursors of secondary PM (Selmi et al. 2016; Torpy et al. 2018). Moreover, plant can prevent the second generation of the particulate matter among atmosphere by reducing the heat island effect of the downtown area (Feyisa et al. 2014; Yang et al. 2017; Salvador et al. 2019). These plant characteristics allow for determination of the air quality through monitoring plant biometrics (Vianna et al. 2011; Perini et al. 2017; Li et al. 2017).

The second is that particulate matter can disrupt plant growth (Shah et al. 2017). In addition to sorting out sunlight, the dust on the leaves blocks the stomata (Hong et al. 2014) and lowers their conductivity to CO_2_ which at the same time hinders photosystem II (Sett 2017).

Finally, food safety is a matter of farm products grown in the air contaminated with particulate matter (Antisari et al. 2015; Zhang et al. 2018). The PM particles adhering to plants include various heavy metals such as Cd and Pb (Puga et al. 2015; Talbi et al. 2018), and it has been confirmed that the concentration varies with the region (Tomašević et al. 2005; Vianna et al. 2011). Heavy metals are toxic to living organisms because of their tendency to accumulate in certain tissues (Auffan et al. 2009; Zhai et al. 2015; Wu et al. 2016; Zhou et al. 2016; Hashim et al. 2017; Li et al. 2017). Air pollution is contributing to the presence of harmful elements, such as, Cd, Hg, and Pb in foods (Matta and Gjyli 2016; Wu et al. 2016). The toxicity of PM components should be assessed at different stages of the food chain (Brandl and Amundson 2008; Exley 2013) to more accurately determine how PM affects the environment and the human health (Baun et al. 2008; Shaheen et al. 2016). The air pollution exposing among the cultivation period of the vegetable threatens the food safety (Lu et al. 2015; Amato-Lourenco et al. 2016; Rai 2016; El-Radaideh and Al-Taani 2018). It has been shown that precipitation during plants growth stage affects the PM residue on leaf surfaces (Little 1973; Sæbø et al. 2012; Xiong et al. 2016; Weerakkody et al. 2018a). Both water-soluble and water-insoluble heavy metals contaminate leaf surfaces (Räsänen et al. 2013). Heavy metal accumulation from consumption of agricultural products is a serious cause of concern in public health (Gidlow 2015; Zhou et al. 2016; Shaheen et al. 2016). Chronic intake of heavy metals negatively affects the human body (Kim et al. 2015a, b; Karri et al. 2016), the effects occurring after years of exposure (Shi et al. 2018).

The purpose of this study is to provide basic data on the safe production of agricultural products, by correlating the location-dependent PM concentration with heavy metal contents in plants.

## Materials and methods

### Research area

**Fig. 1:**
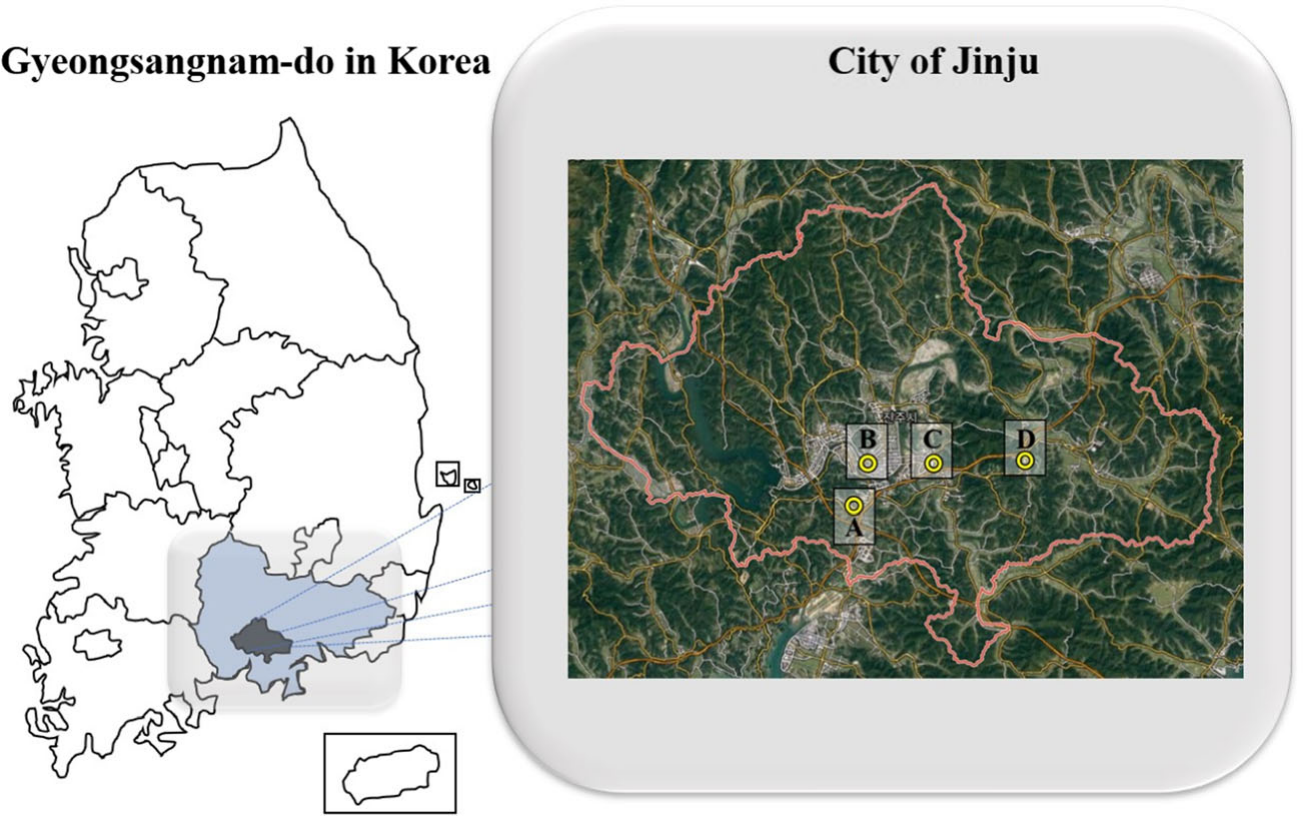
A map showing the location of the study area in Jinju, Gyeongsangnam-do, Republic of Korea: A, Control, Gyeongsang National University (35° 09′ 14.7″ N, 128° 06′ 06.0″ E); B, industrial area (35° 10′ 24.8″ N 128°06′ 04.8″ E); C, near a highway (35°10′ 16.5″ N 128°09′ 43.0″ E); and D, rural area (35°10′ 13.9″ N 128°13′ 28.5″ E)

The study was conducted in four districts of Gyeongsangnam-do, where the cause of PM is expected to be different (Fig. 1). Three outdoor spaces-elementary schools respectively located in an industrial area (Republic of Korea, 35° 10′ 24.8″ N 128° 06′ 04.8″ E), near a highway (35° 10′ 16.5″ N 128° 09′ 43.0″ E), and in a rural area (35° 10′ 13.9″ N 128° 13′ 28.5″ E) and research plant growth chambers at Gyeongsang National University (35° 09′ 14.7″ N 128° 06′ 06.0″ E) as the control, were used. The industrial area used in this study is located directly in a cluster of semi-industrial and general industrial areas, surrounded by small and medium-sized factories. The near-highway area in this study is about 100 m away from the Namhae Expressway. The rural area is an area located within 1-km radius of conservation areas, planning zones, and agricultural areas.

### Cultivation of leafy vegetables

Hydroponic systems were used to grow plants in this study, to prevent soil contaminants from affecting the plants. A rainwater barrier was installed at the base of the plants to minimize the effects of rainwater. On August 20, 2018, *Lactuca sativa* ‘Yeoreumchammatcheokchima’, *Chrysanthemum coronarium* ‘Joongyeop’, and *Spinacia oleracea* ‘Green Top’ were planted in a glasshouse at Gyeongsang National University (Tosilee Medium, Shinan Grow Co, Jinju, Republic of Korea). On September 13, plantlets of these vegetables were placed in the hydroponic systems (model DH-B32, Daesan Precision Co Ltd., Uiwang, Republic of Korea) installed in the three elementary school campuses discussed above and an indoor hydroponics (model DHL-H3, Daes3n Precision Co Ltd., Uiwang, Republic of Korea) installed in the research chamber at Gyeongsang National University. The greenhouse multipurpose nutrient solution was formulated with tap water at Gyeongsang National University’s research greenhouse and transported to each research area for use. After 19 days of cultivation, the plants in the hydroponics were harvested for further analysis.

### Analysis of particulate matter concentration in the research areas

The PM2.5 concentration in the research areas was provided by the Gyeongsangnam-do Office of Education. The concentration was measured by light scattering method. An equipment (Airpro, SGA Embedded Co Ltd., Seoul, Republic of Korea), which installed on the front porch of each elementary school, took a measurement every 6 min.

### Heavy metal analysis

The plants were dried in an oven (FO-450M, Jeio Technology Co. Ltd., Seoul, Republic of Korea) at 70 °C for 72 h. Then, 1.0-g dried samples were ashed in a Nabertherm Muffle Furnace (Model LV 5/11/B180, Lilienthal, Breman, Germany) for 4 h at 525 °C to remove the organic matter. After being treated with 5 mL 25% (*v*/*v*) hydrochloric acid and 10 mL warm deionized water, subsequently, the mixture was filtered to remove ash, and the content of heavy metal elements such as Al, Cd, Cr, Cu, Mn, Pb, and Zn was analyzed with an ICP spectrometer (inductively coupled plasma atomic-emission spectroscopy, ICP-AES, Optima 4300DV/5300DV, PerkinElmer, MA, USA).

### SEM/EDS analysis

Circular shape (0.5 cm in diameter) leaf samples were taken and attached to the specimen stubs with carbon tape. To minimize the loss of PM particles on the leaf surface, the stubs were placed in a stainless-steel Petri-dish and completely dried in the oven at 70 °C. After that, each sample was gold coated (Ion–COATER, SPT-20, COXEM Ltd., Daejeon, Republic of Korea) with a thickness of 15 nm for 200 s under 5 mA. A field emission scanning electron microscope II (SEM/EDS, JSM-7610F, JEOL Ltd., Tokyo, Japan) was used to observe the distribution and size of PM particles on the leaf surface. The distribution points and contents of Al, Cd, Cu, and Pb were analyzed by EDS.

### Statistical analysis

The experiment was set up in a completely randomized design with 8 plantlets per cultivar per replications and 3 replications per treatment. Data collected were analyzed using the SAS statistical software (SAS Institute, Cary, NC, USA). The experimental results were subjected to an analysis of variance (ANOVA) and Duncan’s multiple range tests at *P* ≤ 0.05. Graphs were plotted with OriginPro 2018 program (OriginLab Corporation, Northampton, MA, USA).

## Results and discussion

### Climate and particulate matter concentrations in the research areas

**Fig. 2:**
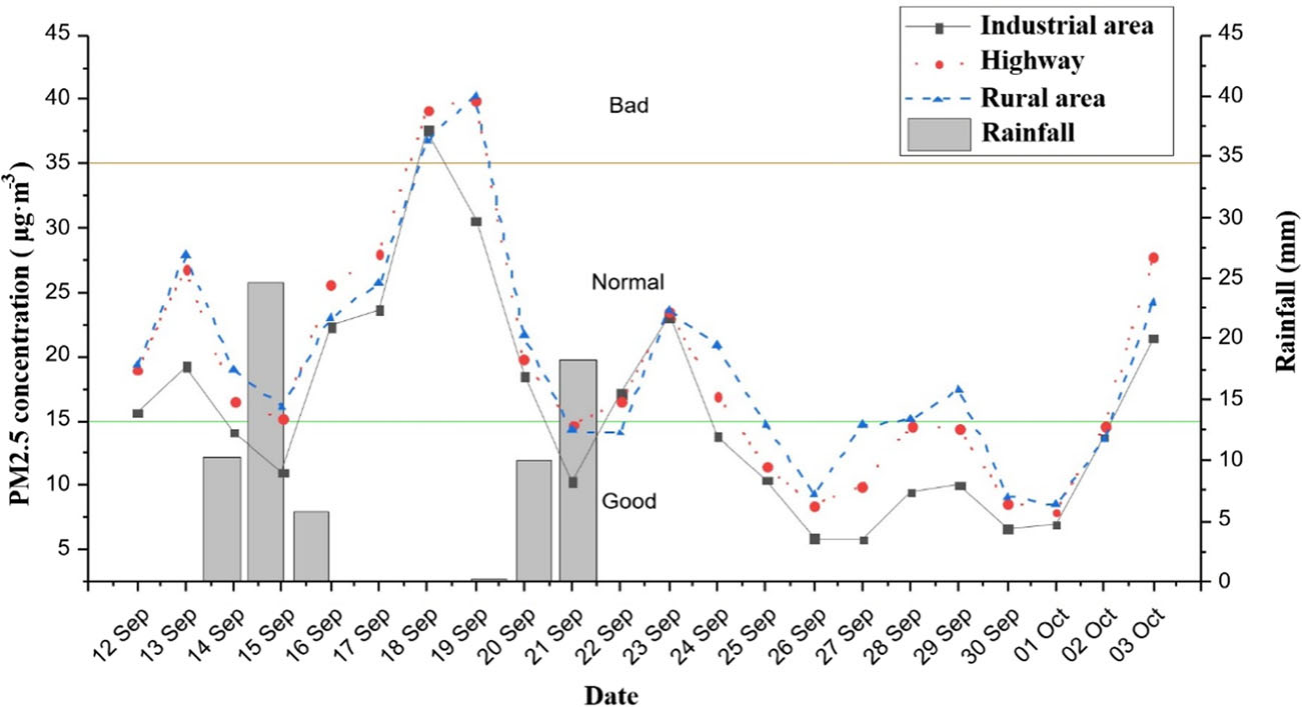
Graphs of the PM concentration and rainfall during the research period. Concentrations of atmospheric PM2.5 were provided from measurements (Airpro, SGA Embedded Co., Ltd., Seoul, Republic of Korea) at each research location. The PM2.5 standards set by the Korean Ministry of Environment are shown in the graph (a value from 0 to 15 μg m^−3^is “good,” from 16 to 35 μg m^−3^ is “normal,” from 36 to 75 μg m^−3^ is “bad,” and 76 μg m^−3^ or higher is “very bad”). Rainfall measurements for the City of Jinju were provided by the Korean Meteorological Administration. Total rainfall for the City of Jinju was 69.3 mm during the research period. It rained for 6 days in total, where the recorded amounts were 10.2 mm (Sep 13), 24.7 mm (Sep 14), 5.8 mm (Sep 15), 0.2 mm (Sep 19), 10.1 mm (Sep 20), and 18.3 mm (Sep 21). Rain was concentrated in the early part of the research period, and there was no rainfall during the second half of the study period. PM trends at all research locations were similar. The average PM concentration in the industrial area was 15.65 μg m^−3^, with the highest value at 48.20 μg m^−3^ (8 pm on Sep 18, 2018); the average PM concentration near the highway was 18.98 μg m^−3^, with 51.50 μg m^−3^ being the highest (7 pm on Sep 19, 2018); the average PM concentration in the rural area was 19.40 μg m^−3^, and the highest value was 49.80 μg m^−3^ (7 pm on Sep 19, 2018)

The City of Jinju, where the research was conducted, is located in Midwest Gyeongsangnam-do, Republic of Korea. It is where the Tongyeong-Daejeon Expressway and the Namhae Expressway are interconnected. The climate is continental, influenced by Jiri-mountain (Choi et al. 2018). The normal temperature in September, when the research was conducted, is between 16 and 27 °C, and the sun is out for 12.5 h per day. Because Jinju is a basin, the temperature difference between night and day is greater than that of many cities of the same latitude (Choi et al. 2018). During the study period, the total rainfall was 69.3 mm, with a maximum rainfall of 24.7 mm and a minimum rainfall of 0.2 mm (Fig. 2).

The average PM2.5 concentration during the total study period was 15.65 μg m^−3^ in the industrial area, 18.98 μg m^−3^ near the highway, and 19.40 μg m^−3^ in the rural area (Fig. 2). The highest PM level was 48.20 μg m^−3^ (20:00, Sep 18, 2018) in the industrial area, 51.50 μg m^−3^ (19:00, Sep 19, 2018) near the highway, and 49.80 μg m^−3^ (19:00, Sep 19, 2018) in the rural area. There was little difference in the PM concentration among the research areas, and the overall PM level trends were similar. None of the research areas experienced “very bad” PM concentrations (Kim 2018) during the study period (Airpro, SGA Embedded Co Ltd., Seoul, Republic of Korea).

### ICP analysis

After being planted and grown in the respective research areas, *Lactuca sativa, Chrysanthemum coronarium*, and *Spinacia oleracea* were harvested. After categorizing new and old leaves on these plants, the internal components were inspected with an ICP spectrometer. The *F* test results revealed significant differences in the concentrations of Al, Cd, Cr, Cu, Mn, Pb, and Zn depending on the cultivar, research area (Antisari et al. 2015; Xiong et al. 2016), and washing conditions (Table 1). Al concentration was high in all plants grown in the industrial area and was the highest in *Chrysanthemum coronarium*.

**Table 1:** This study was conducted in four districts of Gyeongsangnam-do, where the cause of particulate matter contamination is expected to be different: outdoor spaces of three elementary schools, respectively, located in industrial, near-highway, and rural areas, with research plant growth chambers at Gyeongsang National University as the control, were used. The collected plants were divided into two groups to determine the effects of washing on the heavy metal contents of plants. The first group was dried immediately without washing, and the second group was dried after washing. The second group of collected plants was put in tap water. The water was changed twice; then, they were washed for 5 s with running tap water and were completely dried in a 70 °C drying oven for ICP analysis. The ICP spectrometer analyzed for Al, Cd, Cr, Cu, Mn, Pb, and Zn

Species (A)	Area (B)	Wash (C)	Concentration of heavy metals (μg g^−1^DW)				Concentration of heavy metals (ng g^−1^ DW)		
			Al	Cu	Mn	Zn	Pb	Cd	Cr
*Lactuca sativa*	Industrial	Not washed	274.00 b^z^	8.36 lm	162.10 d	62.20 j	1262 i^z^	81 l	400 g
		Washed	42.13 gh	13.36 hij	122.40 h	50.69 o	357 k	150 g	503 f
	Highway	Not washed	26.79 hij	6.54 o	39.28 o	49.60 o	697 l	0 n	363 hi
		Washed	10.42 jk	8.68 l	38.40 o	57.88 lm	2727 a	517 d	453 g
	Rural	Not washed	66.55 fe	7.80 mn	147.08 e	68.39 i	0 q	0 n	379 gh
		Washed	18.40 ijk	19.17 e	126.48 g	57.87 lm	322 k	133 h	541 e
	Growth chambers	Not washed	158.75 c	15.03 f	180.40 c	91.59 g	333 o	25 m	2453 a
		Washed	13.84 jk	19.75 e	210.20 a	61.54 jk	213 mn	813 c	587 d
*Chrysanthemum coronarium*	Industrial	Not washed	319.22 a	13.80 ghi	92.40 j	66.04 i	1905 f	0 n	299 l
		Washed	20.56 ijk	14.69 f	78.93 l	39.92 p	0 q	0 n	127 mn
	Highway	Not washed	50.29 fg	14.90 f	60.79 n	55.32 mn	2379 d	88 l	329 k
		Washed	33.11 hi	23.76 d	58.21 n	66.66 i	459 j	111 i	385 hi
	Rural	Not washed	32.91 hi	10.38 k	68.49 m	59.01 kl	517 n	0 n	163 m
		Washed	121.61 d	45.63 b	85.37 k	54.45 n	667 g	167 f	2667 a
	Growth chambers	Not washed	33.17 hi	19.58 e	134.65 k	80.88 h	358 o	0 n	108 n
		Washed	24.27 ij	38.32 c	164.93 d	81.46 h	240 lm	130 h	707 c
*Spinacia oleracea*	Industrial	Not washed	169.27 c	7.17 on	80.06 l	91.53 g	1157 j	0 n	110 n
		Washed	16.39 ijk	13.24 ij	120.70 h	150.13 b	0 q	0 n	156 lm
	Highway	Not washed	51.21 fg	50.90 a	103.93 i	122.10 e	593 h	0 n	357 ji
		Washed	14.45 jk	10.48 k	59.20 n	122.03 e	257 l	113 i	353 ij
	Rural	Not washed	54.35 fg	7.19 on	77.52 l	107.33 f	80 o	0 n	147 m
		Washed	71.42 e	14.38 gf	119.23 h	144.10 c	847 f	197 e	563 de
	Growth chambers	Not washed	20.74 ijk	13.98 gh	185.43 b	180.80 a	0 q	1800 a	492 e
		Washed	6.22 k	12.90 j	133.97 f	125.27 d	193 n	1420 b	333 j
*F* test	*A*		***	***	***	***	***	***	***
	*B*		***	***	***	***	***	***	***
	*C*		***	***	*	***	***	***	***
	*A* × *B*		***	***	***	***	***	***	***
	*A* × *C*		***	***	***	***	***	***	***
	*B* × *C*		***	***	***	***	***	***	***
	*A* × *B* × *C*		***	***	***	***	***	***	***

***Significant at *P* = 0.001

^z^Mean (*n* = 3) separation within columns followed by different letters are significantly different by Duncan’s multiple range test at *P* ≤ 0.05

**Fig. 3:**
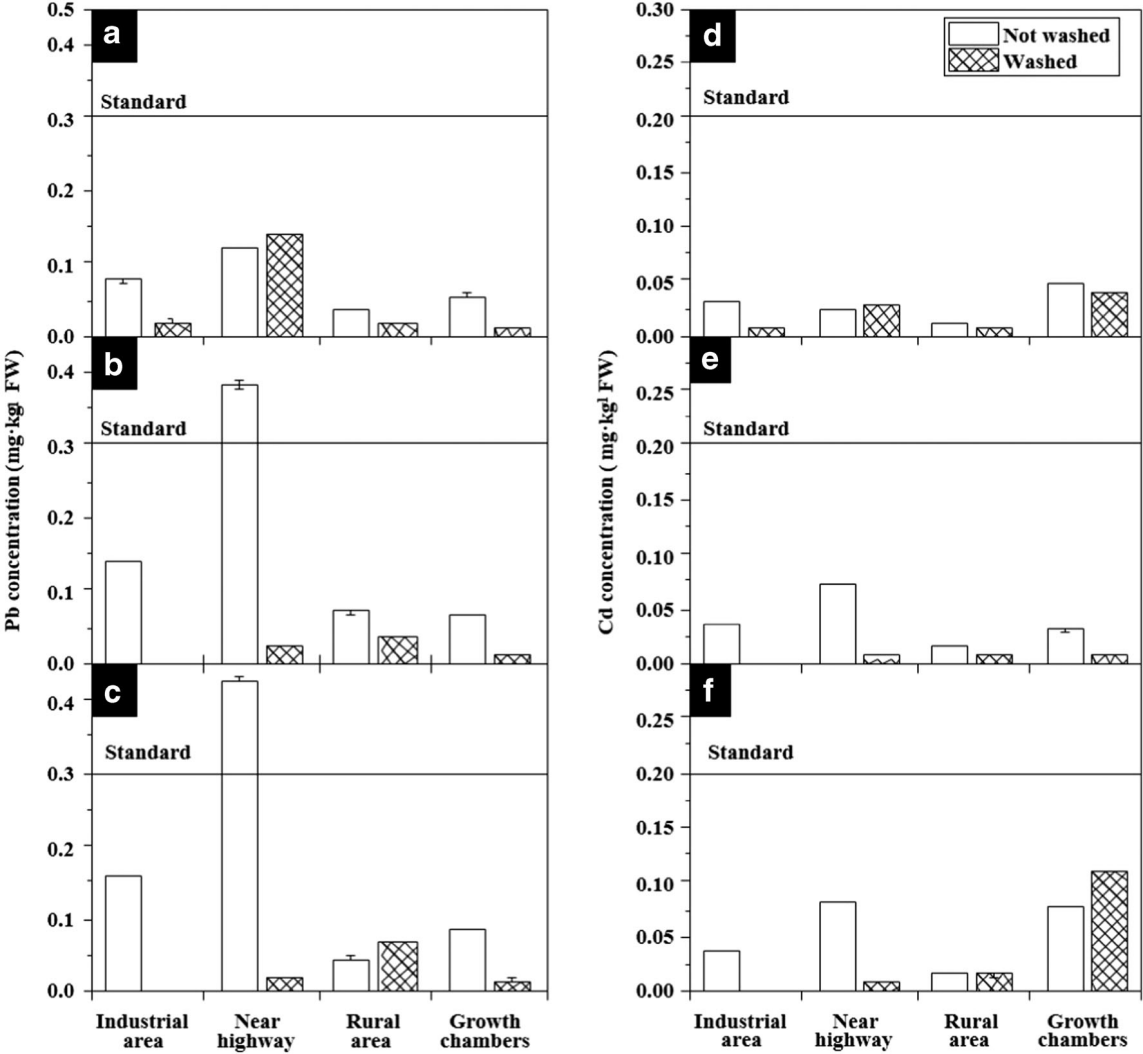
The concentrations of Cd and Pb, among the components identified by the ICP analysis, were compared before and after washing the vegetables, based on the heavy metal standards for leafy vegetables. The graphs above illustrate the following: comparison of Pb concentrations in *Lactuca sativa* by cultivated area, before and after washing (A); comparison of Pb concentrations in *Chrysanthemum coronarium* by cultivated area, before and after washing (B); comparison of Pb concentrations in *Spinacia oleracea* by cultivated area, before and after washing (C); comparison of Cd concentrations in *Lactuca sativa* by cultivated area, before and after washing (D); comparison of Cd concentrations in *Chrysanthemum coronarium* by cultivated area, before and after washing (E); comparison of Cd concentrations in *Spinacia oleracea* by cultivated area, before and after washing (F). The heavy metal standards for leafy vegetables are less than 0.3 mg kg^−1^ of Pb and 0.2 mg kg^−1^ of Cd. The standards are the same for Republic of Korea, Taiwan, EU, and CODEX and are indicated on the graphs. In general, the concentration of heavy metals in leafy vegetables was lower than the standard value. However, Pb concentrations exceeding the standard values were detected in *Chrysanthemum coronarium* (0.383 mg kg^−1^) and *Spinacia oleracea* (0.427 mg kg^−1^) grown near the highway. Overall, the concentration of heavy metals in plants tended to decrease after being washed. Washing with tap water was effective in removing PM particles from the surface of the vegetables

There are two safety criteria for heavy metals in the intake of leafy vegetables: for Cd and Pb (KMFDS 2017). The standards are 0.2 mg∙kg^−1^ for Cd and 0.3 mg∙kg^−1^ for Pb, which are the same for Republic of Korea, Taiwan, EU, and Codex (EC 1997). The ICP data were converted from dry weight to fresh weight for comparison with the safety standards. The Pb levels of vegetables grown near the highway were found to exceed the standard value (Fig. 3). The results are consistent with previous studies showing higher levels of Pb (El-Radaideh and Al-Taani 2018) and higher levels of lead content in plants and soil as close to highways (Hashim et al. 2017; Jankowski et al. 2019).

The contents of accumulated heavy metals were different depending on the plant species (Weerakkody et al. 2018b, c; Zha et al. 2019). It is related to the shape of the leaves, the hair on the leaf surface, and the nature of the wax layer (Weerakkody et al. 2017; Weerakkody et al. 2018b, c; Zha et al. 2019). The Pb concentrations exceeding the standard values were detected in *Chrysanthemum coronarium* (0.383 mg kg^−1^) and *Spinacia oleracea* (0.427 mg kg^−1^) grown near the highway (Fig. 3). Lead is one of the well-known environmental toxins (Gidlow 2015; Karri et al. 2016). Lead causes health problems such as toxicity of the liver, kidneys, hematopoietic system, and nervous system (Kim et al. 2015a, b; Matta and Gjyli 2016). Overall, the concentration of heavy metals tended to decrease before and after washing. Washing with tap water was effective in removing PM particles from the surface.

The composition of PM varies with the region, and even if the PM concentrations on the surface appear similar, the constituents are different depending on the cause (Tomašević et al. 2005; Vianna et al. 2011). Also, even in low concentrations, PM can lead to heavy metal content in plants placed outside for several days (Gajbhiye et al. 2016).

The PM components were also analyzed with respect to the age of the leaves. The elements analyzed for are Al, Cd, Cr, Cu, Mn, Pb, and Zn. The *F* test results showed that the concentration of Al, Cd, Cr, Cu, Mn, Pb, and Zn in plants significantly varies with the plant type, cultivation area, and cleaning conditions (Table 2).

**Table 2:** This study was conducted in four districts of Gyeongsangnam-do, where the cause of particulate matter contamination is expected to be different: outdoor spaces of three elementary schools respectively located in industrial, near-highway, and rural areas, with research plant growth chambers at Gyeongsang National University as the control, were used. The collected plants were divided into two groups according to the leaf age. The plants were completely dried in a 70 °C drying oven before being put analyzed with an ICP spectrometer for Al, Cd, Cr, Cu, Mn, Pb, and Zn

Species (A)	Area (B)	Leaf age (C)	Concentration of heavy metals (μg g^−1^DW)				Concentration of heavy metals (ng g^−1^DW)		
			Al	Cu	Mn	Zn	Pb	Cd	Cr
*Lactuca sativa*	Industrial	Old	274.00 b^z^	8.36 lm	162.10 d	62.20 j	1262 i^z^	81 l	400 g
		Young	154.68 e	14.08 e	51.22 p	92.56 f	1485 g	582 g	691 b
	Highway	Old	26.79 hij	6.54 o	39.28 o	49.60 o	697 l	0 n	363 hi
		Young	10.31 p	2.05 n	12.53 u	44.29 n	2337 d	458 h	281 l
	Rural	Old	66.55 fe	7.80 mn	147.08 e	68.39 i	0 q	0 n	379 gh
		Young	20.23 mn	2.18 n	43.06 q	53.75 l	697 l	253 j	437 f
	Growth chambers	Old	158.75 c	15.03 f	180.40 c	91.59 g	333 o	25 m	2453 a
		Young	7.82 q	10.24 g	42.27 q	120.44 b	1074 k	941 e	511 e
*Chrysanthemum coronarium*	Industrial	Old	319.22 a	13.80 ghi	92.40 j	66.04 i	1905 f	0 n	299 l
		Young	145.30 f	7.37 ij	89.48 h	70.14 h	2721 c	671 f	425 f
	Highway	Old	50.29 fg	14.90 f	60.79 n	55.32 mn	2379 d	88 l	329 k
		Young	16.14 o	3.31 m	21.34 t	54.76 l	7398 a	1371 b	330 k
	Rural	Old	32.91 hi	10.38 k	68.49 m	59.01 kl	517 n	0 n	163 m
		Young	21.83 m	4.78 l	63.84 m	59.69 kj	1343 h	330 i	423 f
	Growth chambers	Old	33.17 hi	19.58 e	134.65 k	80.88 h	358 o	0 n	108 n
		Young	12.21 p	27.63 b	84.88 i	110.66 c	1293 i	586 g	606 d
*Spinacia oleracea*	Industrial	Old	169.27 c	7.17 on	80.06 l	91.53 g	1157 j	0 n	110 n
		Young	108.09 g	4.85 l	58.67 o	96.96 e	2042 e	460 h	347 ijk
	Highway	Old	51.21 fg	50.90 a	103.93 i	122.10 e	593 m	0 n	357 ji
		Young	11.43 p	2.23 n	33.73 s	95.50 e	5477 b	1050 c	283 l
	Rural	Old	54.35 fg	7.19 on	77.52 l	107.33 f	80 p	0 n	147 m
		Young	17.80 no	2.29 n	43.33 q	43.92 n	576 m	235 k	337 jk
	Growth chambers	Old	20.74 ijk	13.98 gh	185.43 b	180.80 a	0 q	1800 a	492 e
		Young	16.65 o	12.35 f	67.96 l	183.39 a	1077 k	1000 d	654 c
*F* test	*A*		***	***	***	***	***	***	***
	*B*		***	***	***	***	***	***	***
	*C*		***	***	***	NS	***	***	***
	*A* × *B*		***	***	***	***	***	***	***
	*A* × *C*		***	***	***	***	***	***	***
	*B* × *C*		***	***	***	***	***	***	***
	*A* × *B* × *C*		***	***	***	***	***	***	***

*NS* non-significant

***Significant at *P* ≤ 0.001

^z^Mean (*n* = 3) separation within columns followed by different letters are significantly different by Duncan’s multiple range test at *P* ≤ 0.05

The Al concentration was high in all plants grown in the industrial area (Mohankumar et al. 2016) and was the highest in *Chrysanthemum coronarium*. According to ATSDR (U.S. Toxic Substance Disorders Registrar General), a small amount of Al accumulated in organ tissues including the bones, brains, and kidneys over a long period of time may damage the human body (Exley 2013; D’Haese et al. 2018). The German Federal Hazard Assessment (BFR) also warns people of eating habits, as daily exposure to Al is more dangerous to children than they are to adults (Nayak 2002; Shrivastava et al. 2018).

It was expected that young leaves would have a lower heavy metal content as they were exposed to PM for a shorter time. However, the content of heavy metals in young leaves was generally higher than that in older leaves (Brandl and Amundson 2008).

Rain was concentrated in the first half of this experiment (Fig. 2). Little (1973) suggested that particle fallout can be more easily removed by heavy rainfall. At this time, many heavy metals attached to old leaves are expected to be washed away (Weerakkody et al. 2018a).

Theoretically, the capacity of foliage accumulation through dry or wet deposition or absorption depends on species features, such as surface area (single leaf and whole foliage), surface texture (roughness and pubescence), plant habitus (evergreen or deciduous), and gas exchange (rate between leaf and atmosphere, multiple stress responses) (Ugolini et al. 2013; Leonard et al. 2016). However, these factors can vary greatly even for the same species in different phases and locations, making the species comparison difficult to be generalized for practical uses (Chen et al. 2016). The leaf waxes were also strongly influenced the accumulation of heavy metal on leaf surfaces. The old leaves and young leaves were equally exposed to PM; however, the results showed that PM stuck more easily to young leaves the old one. A reason may be because wax layers of young leaves were healthier with high density of leaf hairs (Brandl and Amundson 2008). Further research is needed on how the wax layer of the leaves changes with age and how it affects PM adhesion to leaves.

### SEM-EDS

**Fig. 4:**
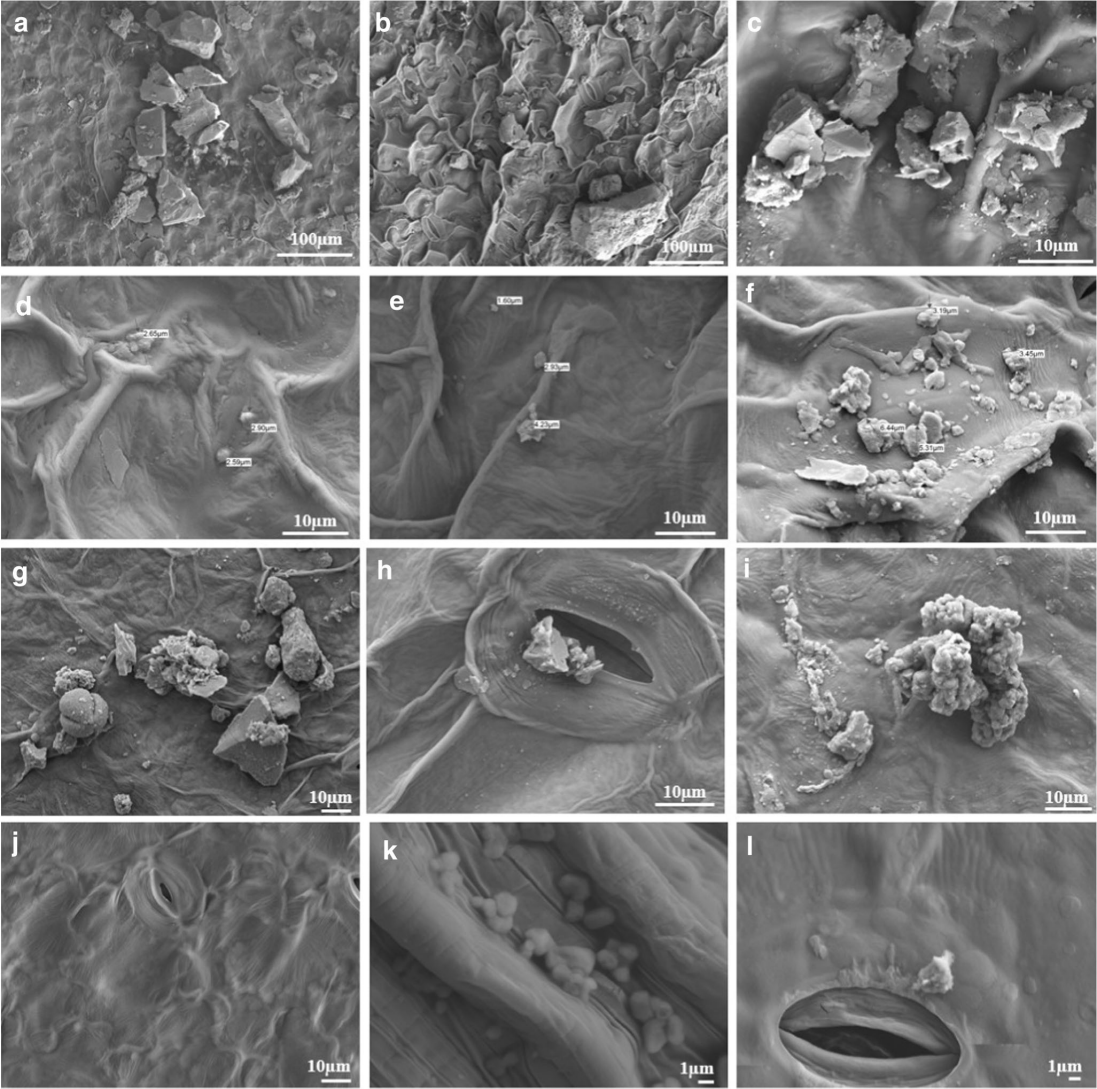
The SEM images of plants used in this study. The plants were dried without washing before the SEM analysis. Plants were grown in an industrial area (**a**–**c**), near the highway (**d**–**f**), in a rural area (**g**–**i**), and in growth chambers (**j**–**l**). **a**, **d**, **g**, **j** are pictures of *Lactuca sativa*; **b**, **e**, **h**, **k** are of *Chrysanthemum coronarium*; and **c**, **f**, **i**, **l** are of *Spinacia oleracea*. Depending on the area where the plants were grown, and on the type of the leafy vegetable, the shape and amount of PM particles attached to the surface are different

The PM particles were observed on the surface of the leafy vegetables (Weerakkody et al. 2018b; Shao et al. 2019). On leaves that have not been cleaned, many PM particles can be observed (Fig. 4). The PM particles found in the growth chambers were under 1 μm in diameter. The air entering the growth chambers does not get filtered separately, but it appears that larger PM particles were blocked during passage through the vents on the roof of the building. However, it is said that nanoscale particles are highly toxic (Jeevanandam et al. 2018), and air circulation systems may be worth installing in plant factories to prevent nanoscale particle pollution (Klaine et al. 2008; Hotze et al. 2010). Pollen particles were also observed on *Lactuca sativa* (Fig. 6g) grown in the rural area. In addition, *Chrysanthemum coronarium* grown in the rural area were observed to have PM particles over their stomata. Through the stomata, PM can enter the plant body (Hong et al. 2014). The shape and distribution of PM particles may vary by region.

**Fig. 5:**
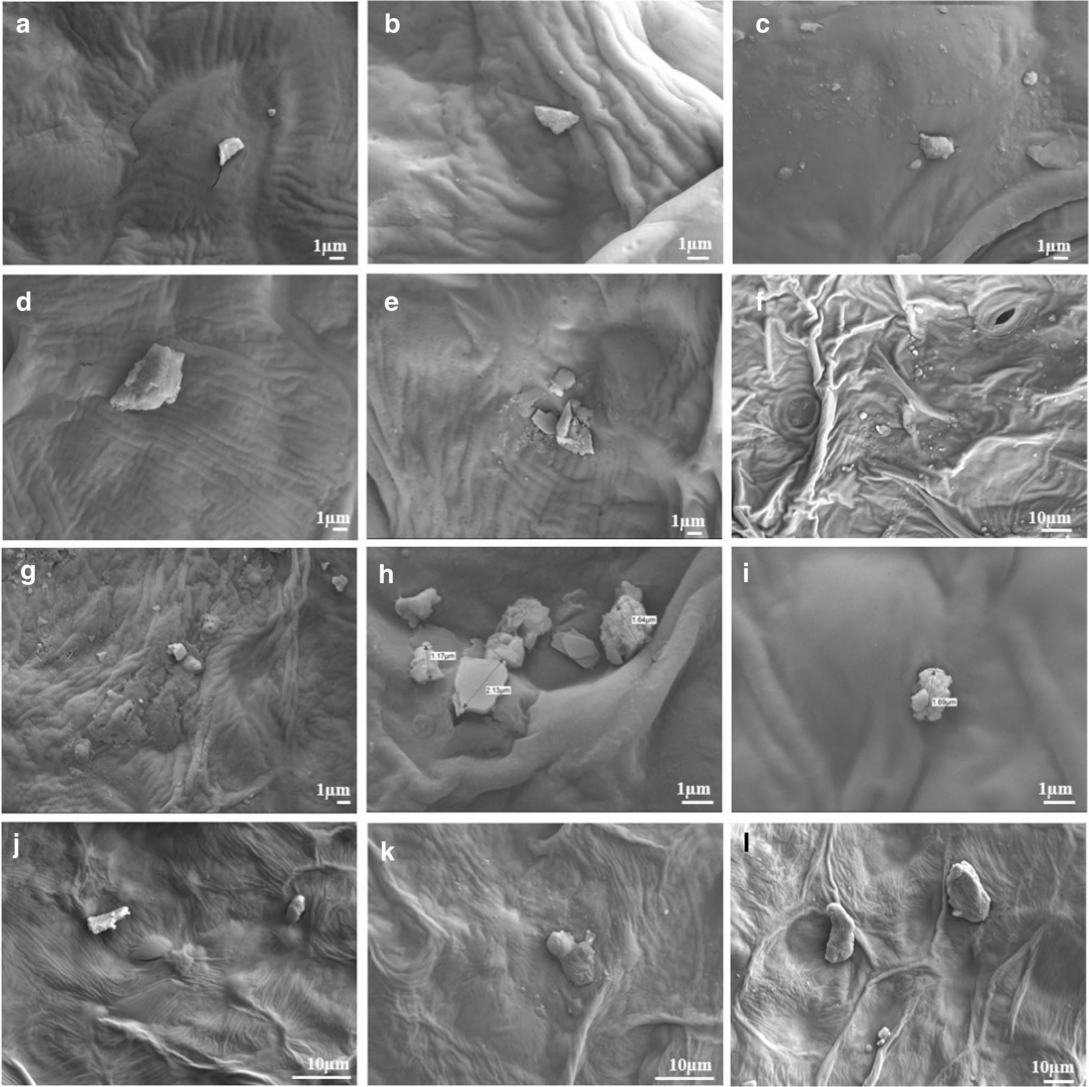
The SEM images of plants used in this study. The collected plants were put into tap water, which was replaced twice. They were then washed with running tap water for 5 s. Afterwards, the plants were dried and imaged with a SEM. **a**–**c** are images of plants grown in the industrial area; **d**–**f** are of those grown near the highway; **g**–**i** are of those grown in a rural area; and **j**–**l** are those grown in growth chambers. *Lactuca sativa* are pictured in **a**, **d**, **g**, **j**; *Chrysanthemum coronarium* in **b**, **e**, **h**, **k**; and *Spinacia oleracea* in **c**, **f**, **i**, **l**. It can be seen that there are less PM particles on the surfaces of washed vegetables compared to those seen on surfaces of plants that were not washed

Upon observation of the surface of the washed vegetables (Fig. 5), it was confirmed that relatively large particles had been removed, but particles around 1 μm remained. This seems to deviate from some trends and may have been caused by the tap water or the washing method used in this study (Table 1). To establish the effects of washing on the PM residue on vegetable surfaces, the study needs to be repeated with a greater number of plants. In addition, various washing methods could not be applied due to the insufficient quantity of the plants used in this study. If this research were to have allowed for various washing methods and their effects to be studied, safer cleaning methods may also have been a finding of this study. Further research is needed on the methods to clean agricultural products, and it will contribute to making consumption guidelines in the situation where consumers’ demand for safe agricultural products is growing (Zhang et al. 2018).

**Fig. 6:**
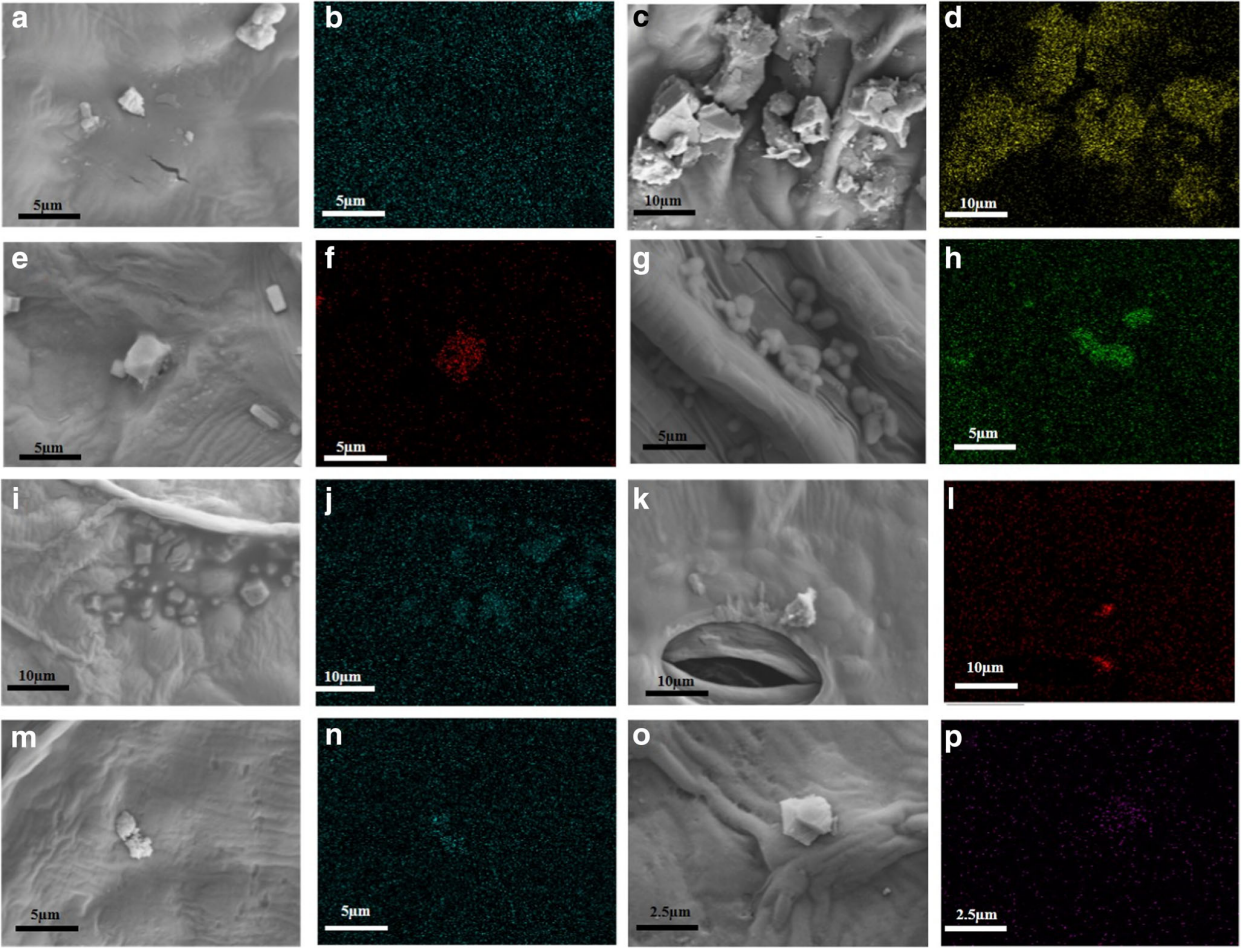
The SEM-EDS was used to identify the remaining ingredients on the surface of leafy vegetables in several samples. The main component found on surfaces of *Lactuca sativa* grown in the industrial area is Pb (**a**, **b**). Al particles are visible on the surface of *Spinacia oleracea* grown in the industrial area (**c**, **d**). There is Na on the surface of *Lactuca sativa* grown in the rural area (**e**, **f**). There is Cd (**g**, **h**) and Pb (**i**, **j**) on the surface of *Chrysanthemum coronarium* grown in the growth chamber. There is Cu on the surface of *Spinacia oleracea* grown in the growth chambers (**k**, **l**). There is Pb on the surface of the washed *Lactuca sativa* grown near the highway (**m**, **n**). Ca is present on the surface of the washed *Lactuca sativa* grown in the rural area (**o**, **p**)

The SEM-EDS was used to identify each component of the PM particles found on leaf surfaces (Fig. 6). Not all components of the particles were verified, but certain components were identified (Lanzerstorfer 2017). Particles of Al, Ca, Cd, Cu, Pb, and Na were identified using EDS (Tomašević et al. 2005). Air pollutants vary with the region (Vianna et al. 2011; Sæbø et al. 2012; Räsänen et al. 2013). It is not possible to identify the composition of PM particles just with the real-time PM concentration information. Therefore, the PM information currently provided by the Republic of Korean government is not sufficient to draw conclusions on food safety (Kim 2018). This is because even at the same concentration, PM pollution is different when Cd and Pb are the main constituents as opposed to when Ca and Na (Fig. 6) are the main components (Auffan et al. 2009). The Cd and Pb are highly toxic metals that are widely present in the environment (Zhai et al. 2015). Many studies have shown that Cd and Pb are transferred to the human body through the food chain (Puga et al. 2015).

## Conclusions

The composition of PM varies depending on the region, and even if PM concentration on the surface appears similar, the components varied depending on the cause. In addition, even if PM is relatively low-concentration, there is a possibility that heavy metals may accumulate in vegetables beyond safe levels if exposure time is extended.

The accumulated heavy metal content varied according to plant species. Since it is related to the shape of the leaves, the hair leaf on the surface, and the properties of the wax layer, a systematic theorem linked to the morphological characteristics of the vegetables eaten is necessary. Various types of heavy metals were detected, such as Al, Cu, Mn, Pb, Cd, and Cr. The concentration of Al was high in all plants grown in industrial areas. The Al can damage the human body with small amounts of Al accumulated in organ tissues, including the bones, brains, and kidneys, over a long period of time. In particular, Pb exceeding safety standards was detected near highways. Lead causes health problems such as the toxicity of the liver, kidneys, hematopoietic systems, and nervous systems. Young leaves were expected to have low heavy metal content due to their short exposure to PM. However, the heavy metal content of young leaves was generally higher than that of older leaves. It is necessary to take a closer look at the accumulation tendency of heavy metals with leaf age. Rain was concentrated in the first half of this experiment. At this time, many heavy metals attached to old leaves are expected to be washed away, and it is necessary to check how much such cleaning can restore the adsorption capacity of particulate matter in terms of the effective way plants remove particulate matter from the atmosphere.

Overall, heavy metal concentrations tended to decrease before and after washing. Washing with tap water has been effective in removing PM particles from the surface.
